# PALLIATIVE CARE FOR PEOPLE WITH MODERATE-SEVERE DEMENTIA IN THE COMMUNITY: RESULTS OF THE IN-PEACE TRIAL

**DOI:** 10.1093/geroni/igae098.2136

**Published:** 2024-12-31

**Authors:** Greg Sachs, Nina Johnson, Sujuan Gao, Minmin Pan, Alexia Torke, Susan Hickman, Kurt Kroenke

**Affiliations:** Indiana University School of Medicine, Indianapolis, Indiana, United States; Regenstrief Institute, Inc., Indianapolis, Indiana, United States; Indiana University School of Medicine, Indianapolis, Indiana, United States; Indiana University School of Medicine, Indianapolis, Indiana, United States; Indiana University School of Medicine, Indianapolis, Indiana, United States; Regenstrief Institute, Inc., Indianapolis, Indiana, United States; Indiana University School of Medicine, Indianapolis, Indiana, United States

## Abstract

Dementia care management programs in the community demonstrate some benefits. Study limitations include limited numbers of people living with dementia (PLWD) with advanced disease or from minoritized populations; lack of palliative care components; and limited success reducing health care utilization. IN-PEACE tested dementia care management integrated with palliative care for PLWD with moderate-severe disease in the community and their caregivers. 201 PLWD-caregiver dyads were randomized to either a dementia care coordinator (99) or usual care (102) and followed for 24 months. Outcomes were neuropsychiatric symptoms (NPI-Q severity) and symptom management (SM-EOLD) in PLWD; distress (NPI-Q distress) and depression symptoms (PHQ-8) in caregivers; and the combined measure of ED visits/hospitalizations. Outcomes were assessed quarterly. Separate mixed effects models were run for each symptom/distress measure and a zero-inflated Poisson model compared the mean number of ED/hospitalization events. Subgroup analyses were conducted based on baseline NPI-Q severity, sex, race, income, and health system. There were no statistically significant differences between groups in any symptoms or distress measures in PLWD or caregivers. PLWD receiving the intervention, however, had substantially fewer ED/hospitalization events (means 1.06 events versus 2.37, p < 0.007). The intervention reduced the proportion of PLWD who had one or more ED/hospitalization events (78.4% of controls vs. 50.5% of intervention, p < 0.001). The relative risk reduction was 35.6% for an event, absolute risk reduction 27.9%, and number needed to treat (NNT) of 3.6. PLWD with higher NPI-Q at baseline and Black PLWD experienced greater reductions in ED visit hospitalization events.

